# Co-occurrence Network Reveals the Higher Fragmentation of the Bacterial Community in Kaidu River Than Its Tributaries in Northwestern China

**DOI:** 10.1264/jsme2.ME17170

**Published:** 2018-05-22

**Authors:** Yang Hu, Chengrong Bai, Jian Cai, Jiangyu Dai, Keqiang Shao, Xiangming Tang, Guang Gao

**Affiliations:** 1 State Key Laboratory of Lake Science and Environment, Nanjing Institute of Geography and Limnology, Chinese Academy of Sciences Nanjing 210008 China; 2 University of Chinese Academy of Sciences Beijing, 100000 China; 3 State Key Laboratory of Hydrology-Water Resources and Hydraulic Engineering, Nanjing Hydraulic Research Institute Nanjing, 210029 P.R. China

**Keywords:** bacterial community, Kaidu River, tributaries, co-occurrence network

## Abstract

Rivers and their tributaries sculpt the earth’s surface, and play an important role in substance circulation and energy flow. Bacteria are involved in most biogeochemical processes in the fluvial ecosystem; however, their pattern distribution in a river and its tributaries has not yet been investigated in detail. In the present study, high-throughput sequencing was employed to examine bacterial communities and their co-occurrence networks between Kaidu River and its nine tributaries in northwestern China. The results obtained demonstrated that both bacterial communities shared a similar dominant sub-community, mainly consisting of *Actinobacteria*, *Bacteroidetes*, and *Proteobacteria*, with *Limnohabitans* and *Variovorax* as the dominant genera. In spite of these commonalities, bacterial community structures still significantly differed between these two habitats, which may be related to the distance-related dispersal limitation. Their co-occurrence networks were generally both positively structured. The structural analysis showed that OTUs from the same phyla were more likely to co-occur. Although the keystone genera were taxonomically different between Kaidu River and its tributaries, they both shared common trophic properties in exploiting niches under oligotrophic conditions. We noted that their relative abundances were less than 1%, indicating the over-proportional roles of rare genera in the bacterial community. In addition, the inferred networks showed less nodes and edges, but higher modularity in Kaidu River than its tributaries, suggesting the higher fragmentation of the bacterial community in the mainstream.

Rivers and their tributaries connect lentic and terrestrial ecosystems. Due to their pivotal roles in the biogeochemical cycle (19, 35), the study of lotic ecology has been attracting increasing interest (24, 42, 54). In attempts to model ecological mechanisms in lotic ecosystems, bacteria are essentially viewed as primary contributors (41, 47). Thus, the dynamics of a bacterial community along a river have received increasing attention. A previous study revealed continuous succession in bacterial community compositions along a river (58). In addition, community richness and evenness were shown to gradually increase from the headwaters to the lower reach (63). Therefore, the distribution of the bacterial community generally fits the expectation of the river continuum concept (RCC), which predicts continua in geomorphological changes and corresponding biotic adjustments along a river (75).

However, a river and its tributaries form not only a longitudinal continuum, but also fluvial networks (7, 11), at which tributaries meet the mainstream. As a consequence, tributary inputs of water, sediment, and particulate organic matter may potentially change the physicochemical properties of the main channel. A tributary source of organisms is concurrently delivered and locally expressed in the river (24, 60, 81). The biota may significantly differ between these two habitats. Clay *et al.* (18) suggested that the density and diversity of macroinvertebrates in tributaries were lower than those in the mainstem. In contrast, the β-diversity of the macroinvertebrate community was markedly higher in tributaries (36). However, few studies have focused on bacterial community compositions between the mainstem river and its tributaries (85). Thus, obtaining a better understanding of the dynamics of the bacterial community in fluvial networks is essential for developing the ecology of riverine ecosystems.

Due to rapid advances in metagenomic technologies, high-throughput DNA sequencing has yielded large amounts of data on bacterial communities (53). A number of analytical methods have also been concurrently proposed in order to enhance our understanding of bacterial communities (45). However, most of these analytical techniques only examine composition and diversity. In order to investigate bacterial communities in more detail, it is crucial to move beyond these basic inventory descriptions. Bacteria live within complex networks through a multitude of interactions (*e.g.* competition, mutualism, and antagonism) (25). However, most of these interactions cannot be directly observed. A co-occurrence network analysis was recently applied to provide important information beyond sample-level comparisons (5, 25, 32). A bacterial network analysis not only reveals how specific species co-occur together, but also identifies the keystone taxa based on their local abundance and habitat specificity. Thus, the construction of a co-occurrence network enables the study of bacterial communities in more detail.

In order to gain novel insights into the relationship between bacterial communities in the main channel and those in its tributaries, Kaidu River and its 9 main tributaries in Northwestern China were selected as a model. The study reach is an alpine cold-arid region with an elevation ranging between 2,734 m and 1,934 m, and the river is typically oligotrophic (40, 66) and sensitive to climate change (9). The channel density is approximately 0.28 kg km^−2^ with an average slope of approximately 12.21%. Its terrestrial landscape is a natural pasture with prohibitive anthropogenic activities, including grassland (60.6%), bare land (20.9%), wetland (8.9%), snow/ice (5.1%), water (3.8%), sand (0.5%), and forest (0.2%) (79). Local meteorological data indicated that the annual air temperature ranges between −26.8°C (January) and 20.4°C (June) (72). The key objectives of the present study were to i) compare bacterial communities in terms of composition and diversity between these two habitats; ii) characterize the architecture of both bacterial co-occurrence networks; iii) elucidate the underlying mechanisms for bacterial community patterns in lotic ecosystems.

## Materials and Methods

### Study area and sampling

**Fig. 1:**
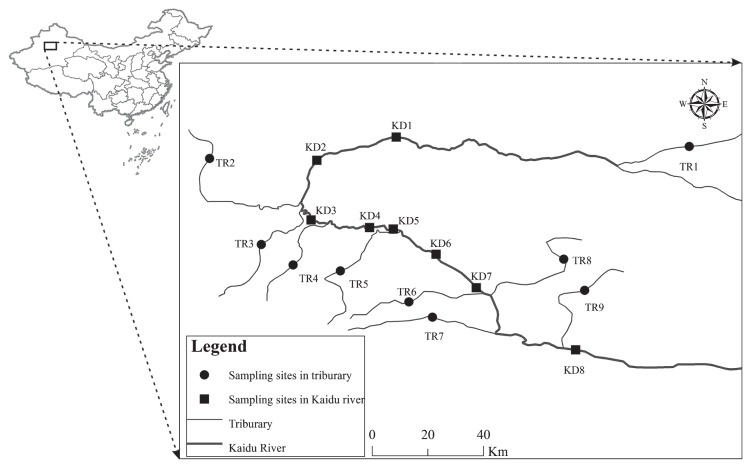
Sampling sites in Kaidu River and its tributaries

Kaidu River (42°14′–43°21′N, 82°58′–86°05′E) is located on the south slope of the Tianshan Mountains in Xiajiang Province in northwestern China, and flows into Lake Bosten. Eight sampling sites (KD1 to KD8) from the river and one sampling site from each tributary (TR1 to TR9) were selected (Fig. 1). Kaidu River is generally fed by meltwater from snow and ice through nine tributaries. A sampling campaign was conducted on 13 and 14 June 2014. Surface water (50 cm) close the riverbank was collected with a 2-L water sampler. Subsamples of 300 mL water for a 16S rRNA gene analysis were then filtered using a 0.2-μm pore-size polycarbonate filter (Millipore) with a hand-driven vacuum pump. The filters were then frozen at −20°C until DNA extraction. The remaining subsample was preserved at 4°C and transported to the laboratory for an immediate chemical analysis.

### Physicochemical analysis

A series of physical parameters, including water temperature, pH, salinity, turbidity, and dissolved oxygen (DO), were assessed *in situ* by a multi-parameter water quality sonde (YSI 6600v2; USA). The concentrations of total phosphorus (TP) and total nitrogen (TN) were evaluated by a colorimetric analysis after digestion (28). The concentration of nitrate (NO_3_^−^N) was analyzed using a Skalar San Analyzer (SAN PLUS, Netherlands). The concentration of total organic carbon (TOC) was assessed by TOC-5000 (Shimadzu) (78).

### DNA extraction, PCR, and Illumina Miseq sequencing

Total DNA for filtered microorganisms was extracted according to Zhou *et al.* (87). Crude DNA extracts were then purified by the E.Z.N.A^®^ cycle-Pure kit (Omega Bio-Tek). The V5–V6 regions of the 16S rRNA genes were amplified using the primers 789F (5′-TAGATACCCSSGTAGTCC-3′; forward primer) and 1068R (5′-CTGACGRCRGCCATGC-3′; reverse primer) (48). Based on the Silva database, the coverage of primers for bacterial communities was 98.0% for both the forward and reverse primers (https://www.arb-silva.de/). Polymerase chain reaction (PCR) amplification was performed in a 50-μL reaction mixture containing 5 μL of 10×PCR buffer, 4 μL of MgCl_2_ (25 mmol L^−1^), 0.5 μL of each primer (10 μmol L^−1^ each), 30 ng quantified template DNA measuring by Pico green, and 0.4 μL of *Taq* polymerase (5 U μL^−1^; Fermentas). PCR cycling was conducted in a thermocycler (Applied Biosystems Veriti Thermal Cycler) by a touchdown program: denaturation at 94°C for 5 min, 11 cycles of denaturation at 94°C for 1 min, annealing at 65°C for 1 min (temperature was decreased by 1°C every cycle until 55°C was reached), and extension at 72°C for 1 min. Nineteen additional cycles were performed at an annealing temperature of 55°C, followed by a final extension at 72°C for 10 min.

Clean amplicon pools for each sample in equal concentrations were paired-end sequenced (2×250) on an Illumina Miseq platform at Personal Biotechnology. (Shanghai, China). Pair-end reads were assembled using FLASH with a minimum overlap of 100 and 8 maximum mismatches allowed in the overlap region (http://www.genomics.jhu.edu/software/FLASH/index.shtml). Briefly, sequencing reads were demultiplexed and filtered for quality and size using the QIIME pipeline (15), denoised with ACACIA (12), and chimeras were identified and removed with ChimeraSlayer (39). Merged sequences were processed to cluster operational taxonomic units (OTUs, 97% identity threshold) by the UCLUST algorithm (29) and classified using the ribosomal database project (RDP) database Release (RDP Release 11.5, http://rdp.cme.msu.edu/) (80). The longest sequence in each cluster was chosen to be the representative sequence, which was annotated according to the SILVA database (30). In order to ensure that rare bacteria were not the result of sequencing errors, we discarded all OTUs with an abundance <0.01% within a sample (67).

### Network construction and characterization

OTUs affiliated with genera were applied to construct the co-occurrence network. Only OTUs that appeared in more than 80% of all samples were considered (88). These previous filtering steps removed poorly represented OTUs and reduced network complexity. Two individual networks within Kaidu River and its tributaries were computed using the CoNet (v1.1.1.beta) plugin within Cytoscape (v3.5.1) as previously described (33, 34). In each network, co-occurrence and mutual exclusion interactions were identified by an ensemble of correlations (Spearman and Pearson coefficients) and distance metrics (Bray-Curtis and Kullback-Leibler dissimilarity measures). One hundred renormalized permutation and bootstrap scores were accessed for each interaction metric and each edge following the ReBoot procedure developed by Faust *et al.* (32). The measure-specific *P*-values from multiple interaction metrics were merged using the Simes method (61) and a false-discovery rate correlation (10) was performed using the Benjamini-Hochberg multiple testing correction. Only 250 top- and bottom-ranking edges from each association measured were kept in the network analysis. The network was examined and visualized using the ‘*igraph*’ package (21). In order to describe the network topology, a set of properties were calculated, including the numbers of nodes and edges, the clustering coefficient, modularity, and betweenness. The algorithm of fast greedy modularity optimization was applied to isolate modules (17, 25). In addition, possible ‘keystone’ OTUs were identified from the co-occurrence network, which were revealed by the high betweenness and closeness score (13).

### Statistical analysis

Before the statistical analysis, all sequences were subsampled with the lowest number of sequences among all sites. All analyses and visualizations were performed by the *vegan*, *geosphere* and *ggplot2* packages in the R environment (version 3.2.2, http://www.r-project.org). The Shannon and Pielou indexes were calculated by the *diversity* function. The Bray-Curtis distance matrix was computed by the *vegdist* function after the Hellinger transformation (45). Non-metric multidimensional scaling (NMDS) was conducted using the *metaMDS* function based on the Bray-Curtis distance. The dendrogram constructed by the UPGMA algorithm was conducted using the *hclust* function. The significance of differences between bacterial communities was tested by PERMANOVA using the *adonis* function. The α-diversity and environmental properties of Kaidu River and its tributaries were compared by Student’s *t*-test through the *t.test* function. The geosphere distance matrix among sampling sites was computed by the *distm* function. The relationship between bacterial community dissimilarities and their spherical distances was tested by the Mantel test though the *mantel* function. The betweenness and closeness values of each node were computed using the *betweenness* and *closeness* functions, respectively (4, 5, 77).

## Results

### Physicochemical characterization of Kaidu River and its tributaries

**Fig. 2:**
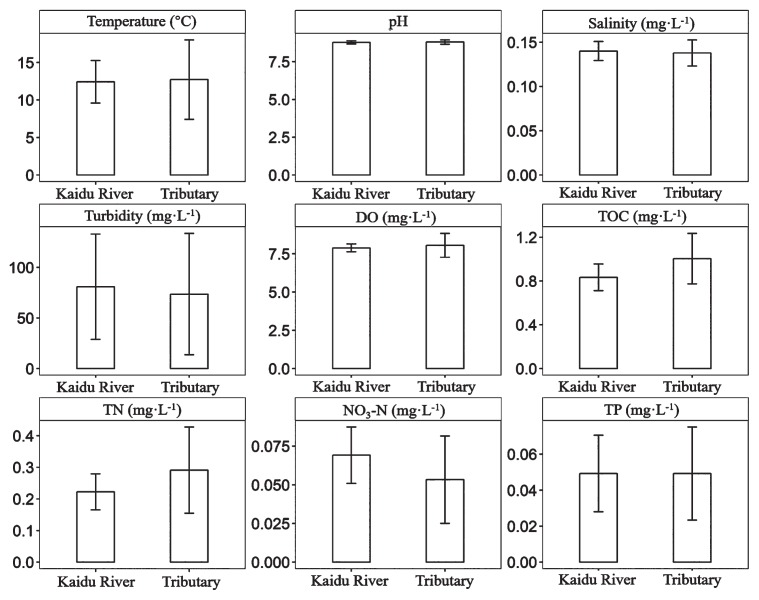
Major physicochemical properties in Kaidu River and its tributaries

No significant differences were observed in major environmental parameters between Kaidu River and its tributaries (the Student’s *t*-test, *P*>0.05, Fig. 2). Temperatures in Kaidu River and tributaries were 12.41±2.84°C and 12.71±2.84°C, respectively. The concentrations of dissolved oxygen ranged between 7.59 mg L^−1^ and 8.29 mg L^−1^ and between 6.61 mg L^−1^ and 9.07 mg L^−1^, respectively. Additionally, no significant differences were observed in chemical properties, including TOC, TN, TP, and NO_3_^−^N (the Student’s *t*-test, *P*>0.05, Fig. 2). These low concentrations indicated the poor nutrient status of these two habitats. All water samples had a pH greater than 8 with the highest value of 9.1, suggesting alkaline conditions in the study area.

### Diversity of bacterial communities in Kaidu River and its tributaries

An average of 42,987 reads was obtained from each sampling site. After trimming, screening, and removing chimeras, 23,809 high quality sequences were obtained on average and were then assigned to 904 and 910 OTUs for Kaidu River and its tributaries, respectively. A total of 873 OTUs were shared by both communities. Good’s coverage was 87.98–96.85%, suggesting that the sequencing effort was sufficient to capture the diversity of bacterial communities (26, 68), and was supported by rarefaction curves, which approached an asymptote (Fig. S1).

**Fig. 3:**
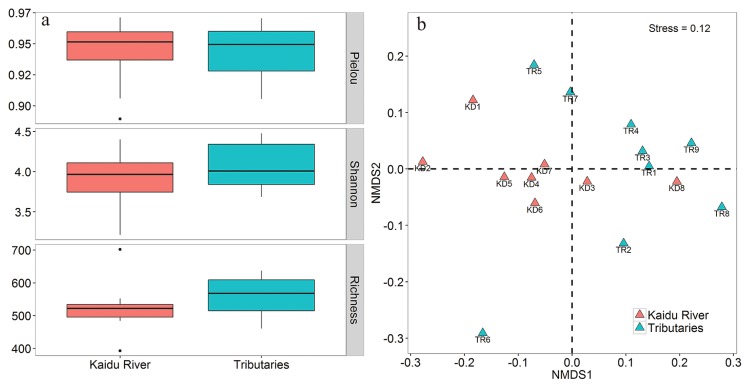
Comparisons of bacterial α-diversity (a) and β-diversity (b) between Kaidu River and its tributaries.

The diversity of bacterial communities in Kaidu river and its tributaries was summarized (Fig. 3). In the case of α-diversity, Richness, Shannon, and Pielou indexes were used as proxies (Fig. 3a). These values were slightly higher in the tributaries than in Kaidu River (the Student’s *t*-test, *P*>0.05). Additionally, NMDS was employed to investigate the β-diversity patterns of bacterial communities (Fig. 3b). Kaidu River was plotted on the left, whereas its tributaries were on the right, suggesting that the bacterial communities in the two habitats were well separated. This result was also supported by the clustering analysis (Fig. S2). The PERMANOVA test also provided a similar result showing that the bacterial community structure was significantly different between Kaidu River and its tributaries (*P*<0.05). Notably, the tributaries were more dispersed than Kaidu River, indicating greater variations in bacterial communities in the tributaries.

### Profiles of bacterial communities in Kaidu River and its tributaries

**Fig. 4:**
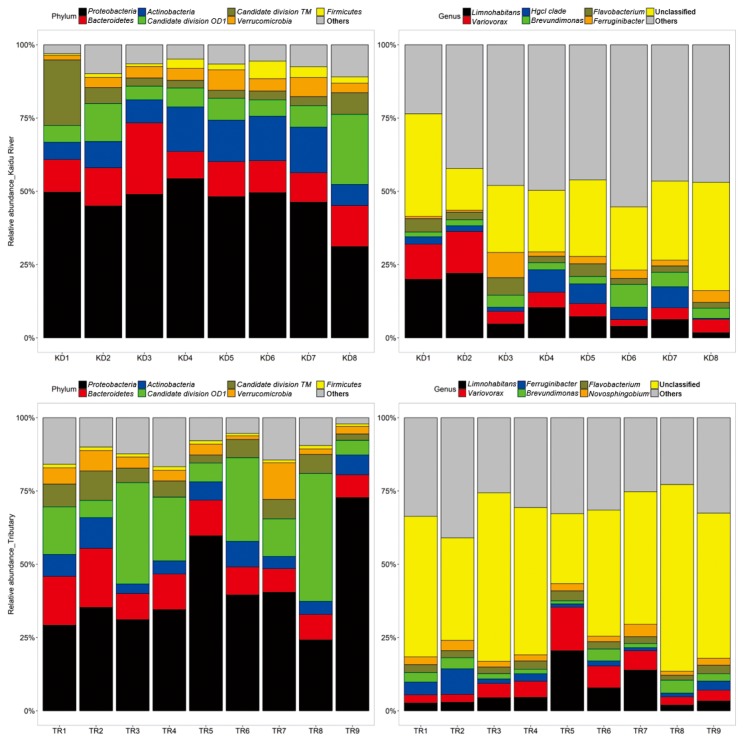
Relative abundance of OTUs at phylum and genus levels in Kaidu River and its tributaries. Only the top seven phyla are shown at each sampling site, and the rest are defined as ‘Others’. At the genus level, only the top six dominants are exhibited. Unknown taxa with significant proportions are grouped as ‘Unclassified’, and the rest are defined as ‘Others’.

After annotating with the SILVA database, the most abundant bacterial phyla in Kaidu River were *Proteobacteria* (51.85%, average relative abundance), *Bacteroidetes* (12.27%), and *Actinobacteria* (11.23%), with *Betaproteobacteria* (26.99%), *Alphaproteobacteria* (21.58%), *Actinobacteria* (10.26%), and *Sphingobacteriia* (5.63%) as the dominant classes. Similarly, bacterial communities in the tributaries shared the same prominent phylotypes: *Proteobacteria* (36.15%), *Bacteroidetes* (12.21%), and *Actinobacteria* (6.31%). *Betaproteobacteria* (23.48%), *Alphaproteobacteria* (9.80%), *Sphingobacteriia* (6.34%), and *Actinobacteria* (5.19%) also dominated as the prominent class. At a finer level, the most abundant genera in Kaidu River and its tributaries were the same: *Limnohabitans* (Kaidu River: 10.80%; Tributaries: 9.00%) and *Variovorax* (Kaidu River: 6.34%; Tributaries: 6.82%) (Fig. 4).

### Architecture of co-occurrence networks in Kaidu River and its tributaries

In the co-occurrence network in Kaidu River, 171 nodes were connected by 186 edges, among which 139 associations were positive. The inference of the bacterial network divided correlating OTUs into 39 modules with a modularity of 0.81 (values >0.4 suggest that the network has a modular structure). The clustering coefficient was 0.28 (how nodes were embedded in their neighborhood). Additionally, 34.29% of co-present OTUs were from the same phyla. The tributaries had a denser co-occurrence network than Kaidu River. The overall network consisted of 160 nodes and 213 edges, among which 191 associations were positive. The network analysis suggested that 25.00% of co-present OTUs were from the same phyla. The high clustering coefficient (0.34) also suggested that the bacterial network was closely connected. The bacterial network was divided into 27 modules with a modularity of 0.74.

**Fig. 5:**
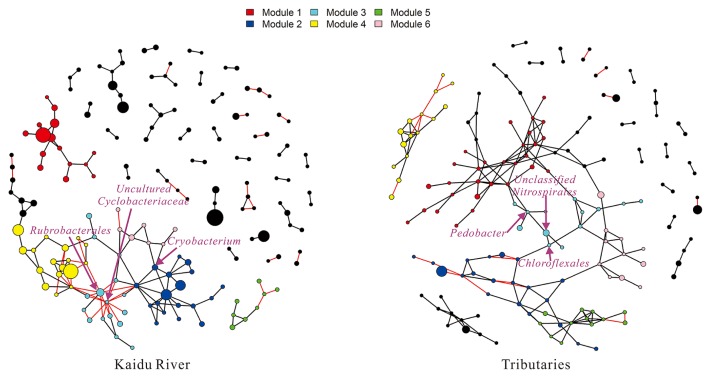
Co-occurrence networks of bacterial communities in Kaidu River. The top 5 modules are presented by different colors. The size of each node corresponds to their relative abundance. Black edges are representative of a positive relationship and red edges of a negative relationship.

Within these two networks, the nodes belonged to 20 and 21 phyla for Kaidu River and its tributaries, with 16 phyla being the same. At a finer level, most of these nodes were affiliated with 26 classes, mainly consisting of *Betaproteobacteria* and *Alphaproteobacteria*. Furthermore, we identified the most influential OTUs within the network as keystone OTUs (Fig. 5). The top three keystone genera of the Kaidu River network were *Rubrobacterales*, *Polynucleobacter*, and *Uncultured Cyclobacteriaceae*. In the case of the tributaries network, the keystone genera were identified as *Chloroflexus*, *Uncultured Nitrospirales*, and *Pedobacter*. Apart from *Polynucleobacter* (1.86%), the relative abundance of other keystone genera was less than 1%.

## Discussion

### Comparison of bacterial communities between Kaidu River and its tributaries

Our results revealed a strong overlap of dominant groups in bacterial communities between Kaidu River and its tributaries. *Proteobacteria*, *Bacteroidetes*, and *Actinobacteria* strongly prevailed in both communities, with *Betaproteobacteria*, *Alphaproteobacteria*, *Actinobacteria*, and *Sphingobacteriia* as the prominent classes. These phylogenetic taxa resembled the dominant groups in other lotic assemblages. *Proteobacteria* (particularly *Betaproteobacteria*), *Bacteroidetes*, and *Actinobacteria* generally dominate the riverine bacterial communities of North America, Europe, and Asia (20, 49, 69, 89). These findings imply a persistent and ubiquitous dominant sub-community in riverine ecosystems. At the genus level, *Limnohabitans* and *Variovorax* were identified as the prominent phylotypes. Their prominence may be due to the high rates of substrate uptake and high mortality rates of bacterivory in nasty habitats (1, 44, 62).

Although Kaidu River and its tributaries shared a similar dominant sub-community, they still showed significant differences in their bacterial community structures. The present results demonstrated that aquatic physicochemical properties were generally similar in both habitats, suggesting a homogeneous environment in the study area. According to traditional paradigms (such as the niche theory and species sorting process), similar bacterial communities were expected between Kaidu River and its tributaries (51, 74). However, our results appear to contradict this expectation. We proposed that the reason may be ascribed to the distance-related dispersal limitation. An increasing body of evidence has highlighted the role of spatial distance on the bacterial community (56, 82, 84). Within Kaidu River, the bacterial community dissimilarity correlated with the geographic distance (Mantel test: *r*=0.70, *P*<0.01, Fig. S2). As they flow downriver, bacterial species are passively transported by unidirectional flow, resulting in source-sink relationships between the communities (46). Consequently, a distance-decay pattern is expected along Kaidu River. In contrast, no correlation was observed between bacterial community dissimilarities and spatial distances in the tributaries (Mantel test: *r*=−0.27, *P*>0.05, Fig. S2). A possible reason for this is the lack of dispersal among distinct tributaries. This is also supported by the rule that distance-related dispersal disappears with an increase in distance on a small-scale (38). Therefore, we concluded that distance-related dispersal may result in differences in bacterial communities between Kaidu River and its tributaries.

### Comparison of co-occurrence networks between Kaidu River and its tributaries

The co-occurrence networks in both habitats were mainly composed of positive correlations. This correlative pattern has been repeatedly documented in the bacterial co-occurrence networks of various habitats. The bacterial community attaching lichen and moss was found to be composed of more than 80% positive correlations (3). Similarly, most microbial species showed positive relationships in different trophic lakes (86). This positive association may be interpreted as cross-feeding, co-colonization, and co-aggregation (32). Thus, these findings suggest a self-structured and self-sustaining assortment of bacterial communities (6, 8). Moreover, a previous study demonstrated that these positive correlations, in turn, correlated with phylogeny (83). Although we did not observe this relationship, our results still implied that large proportions of positive correlations consisted of OTUs from the same phyla (34.29% and 25.00% for Kaidu River and its tributaries, respectively). This is consistent with previous findings. For example, 33% of OTUs from the same phyla were more likely to co-occur in soil bacterial communities (6). This pattern may occur because phylogenetically close species have stronger mutualistic correlations (55), and these are due to their similar habitat preferences and niche adaptations (27, 71, 73).

Highly connected nodes in the co-occurrence network are generally analogous to the keystone genus (37, 70). The most connected nodes of the Kaidu River network were representatives of *Rubrobacterales*, *Polynucleobacter*, and *Uncultured Cyclobacteriaceae*. *Rubrobacterales* appear to play pivotal functions in extreme environments (*i.e.* low nutrient levels and low productivity), such as biomineralization (22, 59). *Polynucleobacter* are potential primary producers in the oligotrophic environment (52, 57). As the most keystone genus in the tributaries network, *Chloroflexus* have been reported as typical phototrophs that contain bacteriochlorophyll *a* (BChl *a*) (50). As the sub-keystone genus, *Pedobacter* have been suggested to play an important role in the decomposition of organic matter and nutrient dynamics (64, 65).

In spite of taxonomical differences in these keystone genera within the two networks, they all share common trophic properties in exploiting ecological niches under oligotrophic conditions. Additionally, we noted that most of these keystones were less than 1%, suggesting the significance of rare genera in bacterial communities. Rare genera are being increasingly recognized as crucial components of communities in biochemical processes and community assembly (14, 43, 76). However, given the challenges associated with investigating rare microbes and rapid advances in experimental approaches, further studies are needed on rare microbes.

The present results demonstrated that the Kaidu River network was less complex and less coherent than its tributaries. The number of nodes (171) and edges (186) in the network from Kaidu River were less than in its tributaries (160 nodes and 213 edges). Additionally, the network in Kaidu River showed higher fragmentation (39 modules and 0.81 modularity) than its tributaries (27 modules and 0.74 modularity). Despite the small number of similar studies with which to compare, a recent study reported similar findings to the present results (81). We postulated mechanisms linked to metacommunity dynamics and biodiversity, as supported by theoretical and empirical evidence (2, 16, 31), to drive the differences in co-occurrence networks between Kaidu River and its tributaries. According to the concept of metacommunity (46), bacterial communities in the lotic ecosystem are partially driven by a common terrestrial origin of aquatic communities (60). Due to the constrained contributing area, the metacommunity size of tributaries was larger than Kaidu River. Besemer *et al.* (11) showed that these effects may increase biodiversity in tributaries, as demonstrated by the present results (Fig. 3). In turn, higher biodiversity promotes interactions in bacterial communities (23) and also increases their co-occurrence pattern (lower fragmentation) (81). Thus, Kaidu River showed higher fragmentation than its tributaries.

## Conclusion

The present study provides insights into bacterial communities in Kaidu River and its tributaries. Our results demonstrated that although Kaidu River and its tributaries shared a similar dominant sub-community, there were also significant differences in bacterial community structures between the two habitats. Furthermore, we constructed their bacterial co-occurrence networks. The results obtained showed that most correlations in the networks were positive. Additionally, the structural analysis showed that OTUs from the phyla were more likely to co-occur. Within Kaidu River, the keystone genera were *Rubrobacterales*, followed by *Polynucleobacter* and *Uncultured Cyclobacteriaceae*. In the case of tributaries, the most keystone genera were identified as *Chloroflexus*, *Uncultured Nitrospirales*, and *Pedobacter*. Although these keystone genera were phylogenetically different between Kaidu River and its tributaries, they all showed similar functions under oligotrophic conditions. Notably, their rare proportions highlighted the importance of rare genera in bacterial communities. In addition, the inferred networks showed less nodes and edges, but higher modularity in the Kaidu River network than in the tributaries network. Ultimately, the co-occurrence network analysis revealed higher fragmentation in the bacterial community of the mainstream than its tributaries.

## Supplementary Material
