# Supplementary material for: Co-occurrence Network Reveals the Higher Fragmentation of the Bacterial Community in Kaidu River Than Its Tributaries in Northwestern China
Source: Microbes Environ. 2018 May 22;33(2):127–34. doi: 10.1264/jsme2.ME17170 (PMC6031398; doi:10.1264/jsme2.ME17170)
Supplement: Supplementary file 1 [file 33_127_s1.pdf]

Co-occurrence network reveals a higher fragmentation of the bacterial community in Kaidu River than its tributaries in Northwestern China

Yang Hu[1, 2], Chengrong Bai[1,2], Jian Cai[1,2], Jiangyu Dai[3], Keqiang Shao[1], Xiangming Tang[1], Guang Gao[1,*]

[1] State Key Laboratory of Lake Science and Environment, Nanjing Institute of Geography and Limnology, Chinese Academy of Sciences, Nanjing 210008, China

[2] University of Chinese Academy of Sciences, Beijing, 100000, China

[3] State Key Laboratory of Hydrology-Water Resources and Hydraulic Engineering, Nanjing Hydraulic Research Institute, Nanjing, 210029, P.R. China

Running headline: Bacterial community in lotic ecosystems

[*] Corresponding author: Guang Gao, Nanjing Institute of Geography and Limnology, Chinese Academy of Sciences, 73 East Beijing Road, Nanjing 210008, China
E-mail: guanggao@niglas.ac.cn
Phone: (+86) 25 86882187
Fax: (86) 25 57714759

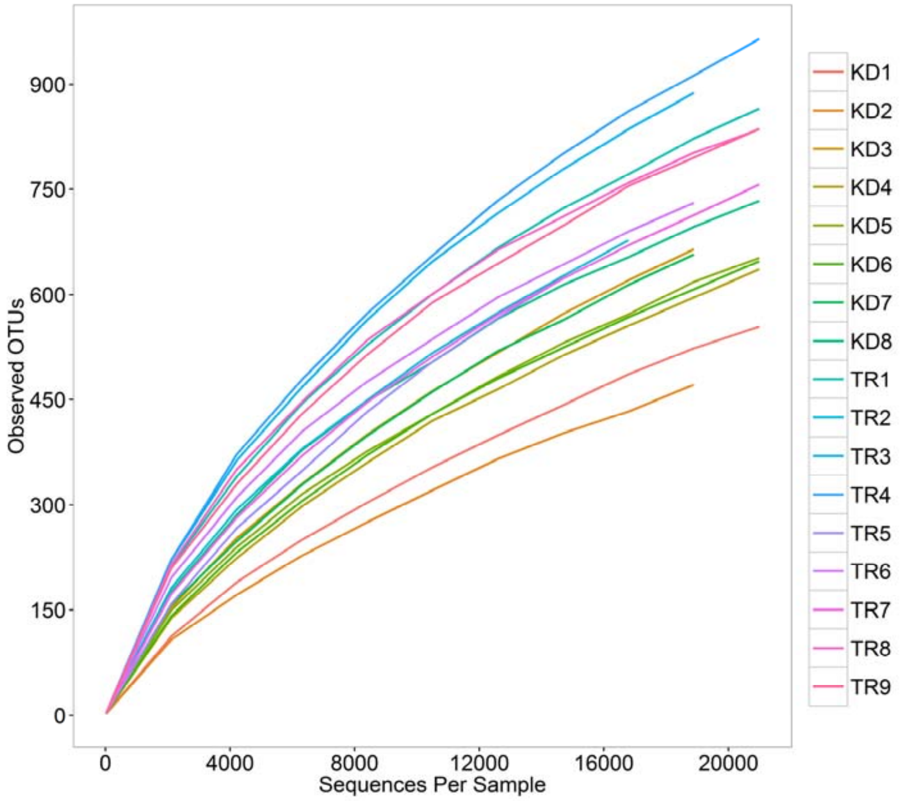

Fig. S1 The rarefaction curve of bacterial community in the Kaidu River and its tributaries. KD represents Kaidu River, TR represents its tributaries

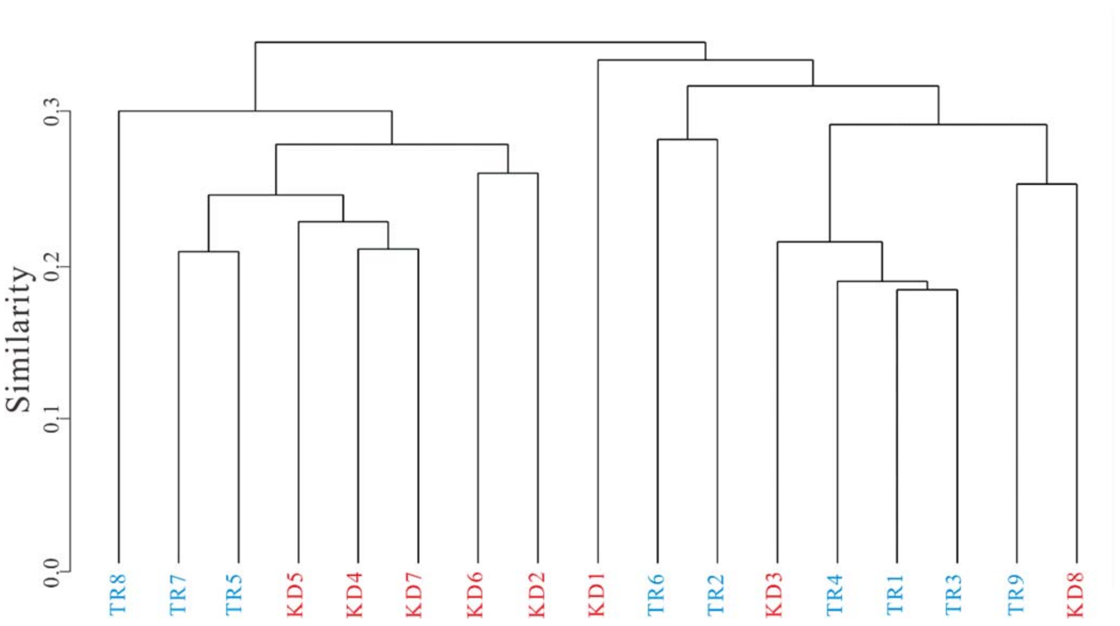

Fig. S2 The dendrogram of the bacterial communities in the Kaidu River and its tributaries based on UPGMA algorithm

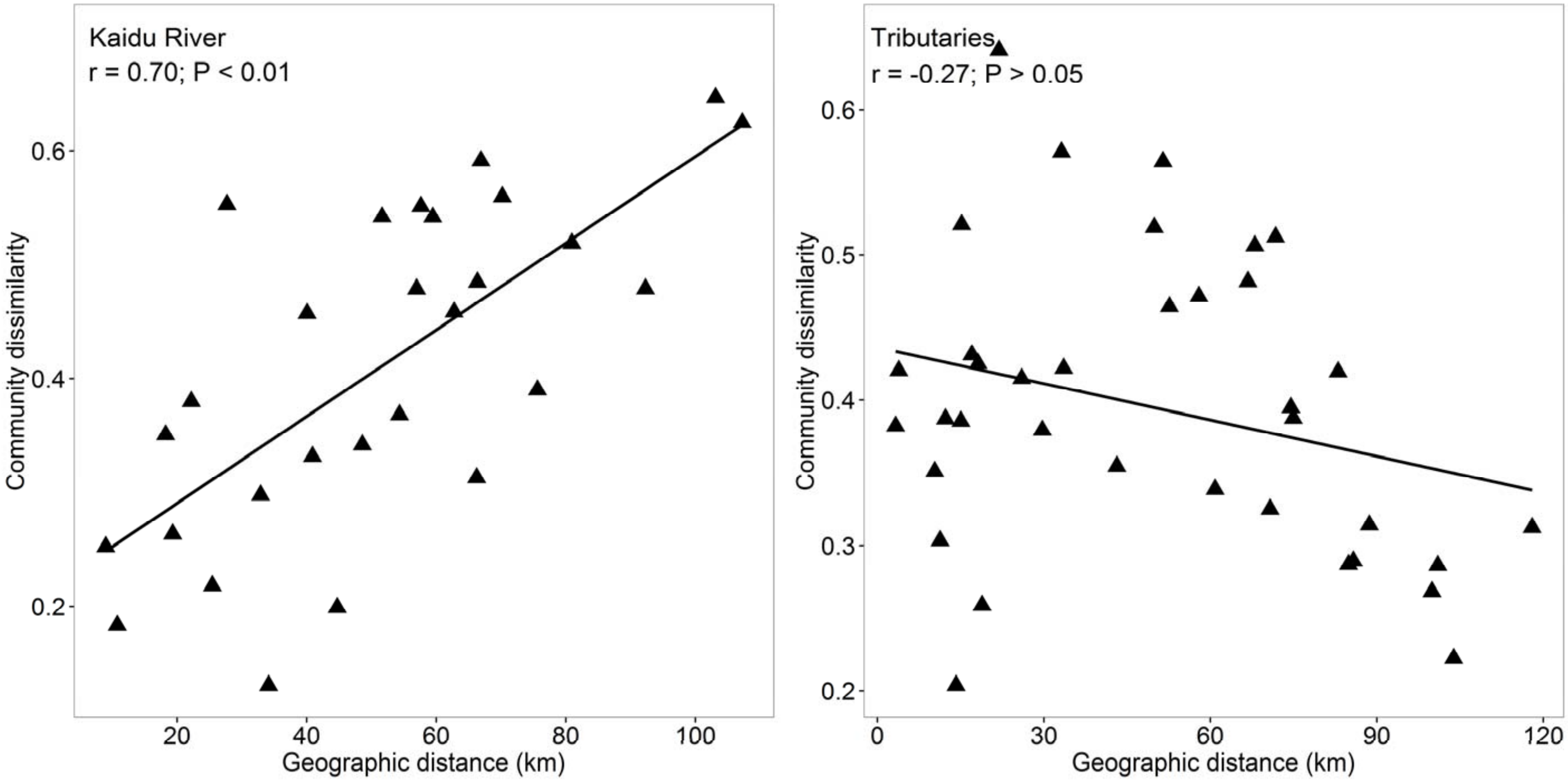

Fig. S3 The relationships between Bray-Curtis dissimilarity and geographic distance in the Kaidu River and its tributaries
